# Exercise regulates shelterin genes and microRNAs implicated in ageing in Thoroughbred horses

**DOI:** 10.1007/s00424-022-02745-0

**Published:** 2022-09-09

**Authors:** Shama Mandal, Michele M. Denham, Sarah J. Spencer, Joshua Denham

**Affiliations:** 1 School of Health and Biomedical Sciences, RMIT University, Melbourne, VIC Australia; 2 Jubilee Stud, Freshwater Creek, Victoria, Australia; 3 ARC Centre of Excellence for Nanoscale Biophotonics, RMIT University, Melbourne, VIC Australia; 4 School of Health and Medical Sciences, University of Southern Queensland, Ipswich, QLD 4305 Australia; 5 School of Science and Technology, University of New England, Armidale, NSW Australia

**Keywords:** Telomere, *TERT*, Non-coding RNA, miRNA, Equine

## Abstract

Ageing causes a gradual deterioration of bodily functions and telomere degradation. Excessive telomere shortening leads to cellular senescence and decreases tissue vitality. Six proteins, called shelterin, protect telomere integrity and control telomere length through telomerase-dependent mechanisms. Exercise training appears to maintain telomeres in certain somatic cells, although the underlying molecular mechanisms are incompletely understood. Here, we examined the influence of a single bout of vigorous exercise training on leukocyte telomerase reverse transcriptase (*TERT*) and shelterin gene expression, and the abundance of three microRNAs (miRNAs) implicated in biological ageing (miRNA-143, -223 and -486-5p) in an elite athlete and large animal model, Thoroughbred horses. Gene and miRNA expression were analysed using primer-based and TaqMan Assay qPCR. Leukocyte *TRF1*, *TRF2* and *POT1* expression were all significantly increased whilst miR-223 and miR-486-5p were decreased immediately after vigorous exercise (all *p* < 0.05), and tended to return to baseline levels 24 h after training. Relative to the young horses (~ 3.9 years old), middle-aged horses (~ 14.8 years old) exhibited reduced leukocyte *TERT* gene expression, and increased *POT1* and miR-223 abundance (all *p* < 0.05). These data demonstrate that genes transcribing key components of the shelterin-telomere complex are influenced by ageing and dynamically regulated by a single bout of vigorous exercise in a large, athletic mammal — Thoroughbred horses. Our findings also implicate *TERT* and shelterin gene transcripts as potential targets of miR-223 and miR-486-5p, which are modulated by exercise and may have a role in the telomere maintenance and genomic stability associated with long-term aerobic training.

**Supplementary Information:**

The online version contains supplementary material available at 10.1007/s00424-022-02745-0.

## Introduction 

Ageing is a complex biological process involving the progressive loss of bodily functions and degradation of serial, repeated DNA located at the end of chromosomes — the telomeres. The finite capacity of cells to divide is ultimately determined by telomere length, since excessive shortening triggers cellular senescence [8, 22]. Relatively short telomeres are associated with common age-related conditions (e.g. heart disease) and severe telomere shortening underpins rare diseases with poor longevity, known as telomeropathies [21]. Age-related telomere shortening and the deletion of genes that protect telomeres culminates in accelerated telomere attrition and reduced life expectancy. Furthermore, telomere-lengthening therapies increase organism vitality and improve longevity. Therefore, telomere shortening is considered a key hallmark of ageing [25].

The rate of telomere shortening is predictive of lifespan, which is an observation consistent amongst vertebrates [51]. It also seems to be affected by psychological stress and exercise training. Whilst the former is associated with accelerated telomere shortening [43], those regularly active or engaged in endurance training possess longer leukocyte telomeres than inactive controls [14]. In rodents, long-term exercise training attenuates age-related telomere shortening in the heart and aorta [27, 49, 50]. Although many of the molecules governing telomere length are known, their roles in exercise-induced telomere maintenance are unclear.

Telomere integrity is protected by six shelterin proteins. Telomeric repeat binding factor (TRF) 1 and 2 form homodimers and preferentially bind to double-stranded telomeric DNA. TRF1 interacting nuclear factor 2 (TINF2) binds and interacts with TRF1/TRF2 and ACD shelterin complex subunit and telomerase recruitment factor (ACD) [34], which has a crucial role in telomerase recruitment to the telomeres [37]. TRF2 interacting protein (TRF2IP) associates with TRF2. Finally, protection of telomeres 1 (POT1) binds directly to single-stranded telomeric DNA and double-stranded telomeres through interactions with ACD [39]. All shelterin proteins share crucial roles in telomere protection by preventing DNA damage response pathways and control telomere length by recruiting, impairing and/or regulating telomerase [3, 32, 39].

Notably, telomerase extends telomeres thereby attenuating the age-associated attrition caused by cell division (i.e. the end-replication problem). Telomerase activity is up-regulated by a single bout of exercise and after chronic long-term exercise training [11]. Furthermore, the major protein and rate-limiting component of the telomerase enzyme, telomerase reverse transcriptase (*TERT*), gene expression is increased in peripheral leukocytes 1 h after a single bout of exercise and chronic training in human whole blood and peripheral blood mononuclear cells (PBMCs) [11]. Long-term aerobic training is also associated with increased whole blood leukocyte *TERT*, as exhibited by endurance athletes compared to healthy controls [12]. Telomere length and integrity are ultimately regulated by shelterin and interactions with telomerase. However, the molecular mechanisms governing shelterin, *TERT* expression and telomerase activity and how they are affected by exercise are incompletely understood.

Small RNA molecules, such as microRNAs (miRNAs), have been implicated in biological ageing and the regulation of telomerase and shelterin. MiRNAs are evolutionary conserved small (18–25-base long) RNA molecules that bind to mRNAs via complete or partial sequence complementarity, and typically reduce protein translation and mRNA stability [46]. MiRNAs interact with specialised proteins to form an RNA-induced silencing complex and bind to the 3′ untranslated region (UTR) of target mRNAs to hinder protein translation and promote mRNA instability [18]. They also bind to mRNA coding sequences, yet these are less effective at impeding protein translation than those binding to 3′UTRs. Although many miRNAs are regulated by short- and long-term exercise training in numerous tissues [13, 41], analyses involving the measurement of miRNAs in context with shelterin and *TERT* gene expression are scarce.

To examine if the molecular mechanisms governing shelterin and *TERT* gene expression are affected by exercise, we used a large animal model of excellent aerobic fitness. Our previous work established that telomere length is inversely related to age in Thoroughbred horses [9] and that telomeres in these large mammals are much more similar in length to that of humans than in small animal models, such as rodents [15, 20]. Unlike mice and rats, but like humans, telomerase activity is very low or absent in somatic cells from large animals. Using this Thoroughbred racehorse model, we determined the influence of vigorous exercise training on leukocyte shelterin gene expression. To understand the molecules regulating changes in shelterin gene expression in a large athletic animal model after exercise, we also analysed three miRNAs previously implicated in biological ageing (miR-143, miR-233 and miR-486-5p) [42, 44], those that also target key shelterin mRNA according to the miRWalk database. Finally, we examined the resting expression of shelterin genes and miRNAs of young horses currently in training for racing and compared them to retired middle-aged horses not performing structured aerobic training to assess the proximal effect of exercise on telomere integrity.

## Results

### Exercise session

**Fig. 1:**
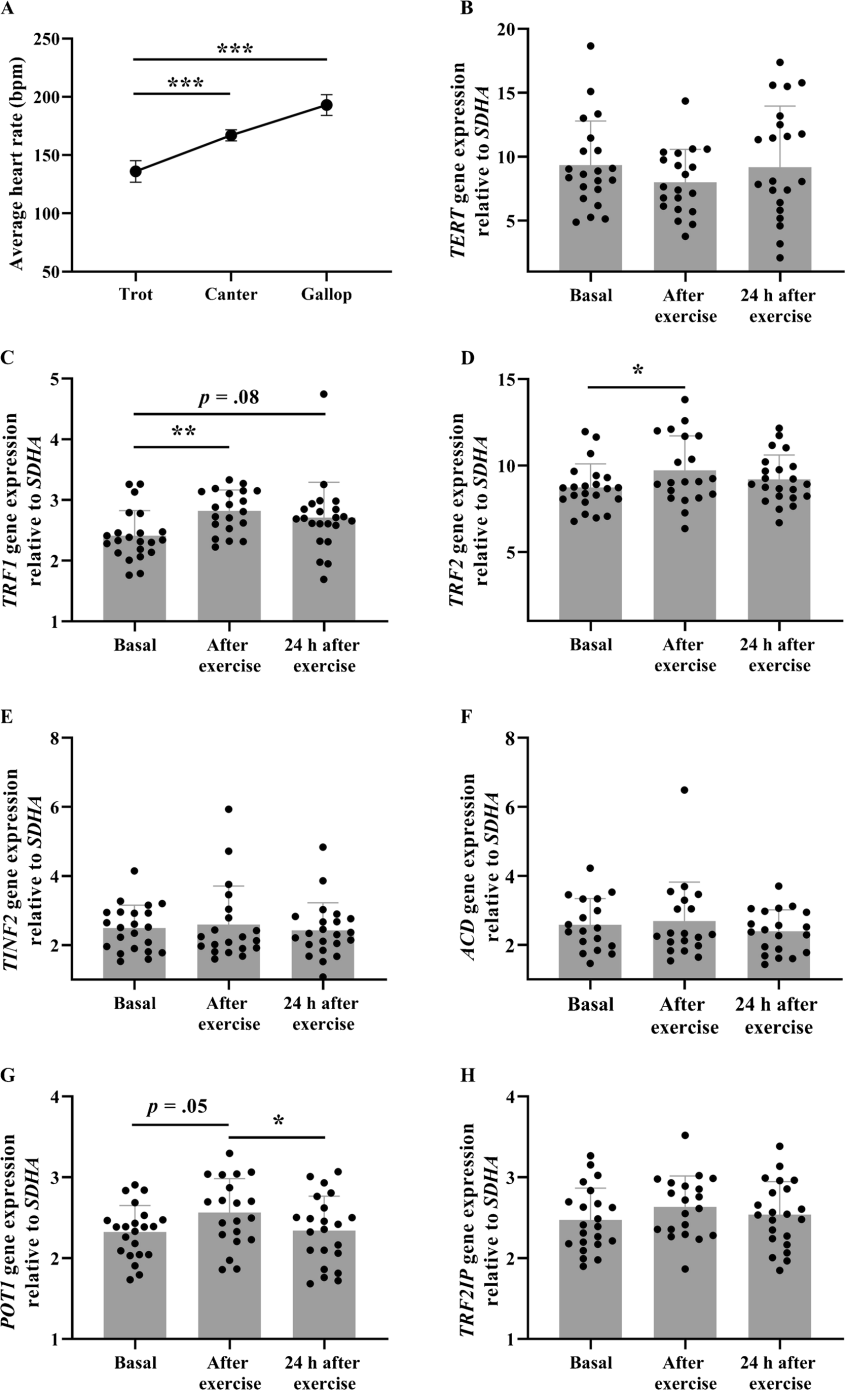
Leukocyte telomere regulating gene expression changes after exercise in Thoroughbred racehorses. **A** Thoroughbred racehorse exercise training and heart rate responses (*n* = 10–13). The average heart rate of the horses progressively increased with the elevated training intensities (*p* < 0.001). Data are from a mixed-effect model. *TERT* (**B**) and shelterin (**C–H**) gene expression changes immediately after and 24 h after vigorous exercise training in Thoroughbred horses (*n* = 17–22). Data are mean ± SD relative gene expression. *p < 0.05, **p < 0.01, ***p < 0.001

The characteristics of horses in the exercise session are outlined in Supplementary Table 3. GPS and heart rate data from the typical ‘workday’ exercise training session are displayed in Supplementary Table 4. On average, the session duration was 616 ± 40.5 s and horses reached peak speeds of 60.3 ± 1.1 kph during the gallop phase. The horses’ average heart rate during exercise training increased significantly at each training intensity — trot, canter and gallop (heart rate [bpm] mean ± SE: 136 ± 9.31 to 167 ± 4.8 to 193 ± 8.99, respectively, overall main effect *p* < 0.001; Fig. 1A). The average peak heart rate during the exercise session was 221 ± 2.6 bpm (Supplementary Table 4), indicating that the exercise was high-intensity training, as heart rates were close to maximal for trained Thoroughbred racehorses [4, 35].

### Whole blood leukocyte TERT and shelterin gene expression after exercise training

Whole blood leukocyte telomere-regulating gene expression before, immediately after and 24 h after exercise are displayed in Fig. 1B-H and Supplementary Table 5. *TRF1* was up-regulated immediately after exercise (*p* = 0.0035) and tended to remain elevated at 24 h, although this result was borderline statistically significant (*p* = 0.08; Fig. 1C). *TRF2* was also up-regulated immediately after exercise (*p* = 0.04; Fig. 1D), and returned to pre-exercise levels 24 h later. Similar to *TRF2*, *POT1* tended to be up-regulated immediately after exercise (*p* = 0.05) and returned to pre-exercise levels 24 h later (Fig. 1G). No marked differences were observed at any of the time points for *TERT*, *TINF2*, *ACD* or *TRF2IP* (all *p* > 0.05; Fig. 1B, E, F  or H and Supplementary Table 5).

### Whole blood leukocyte TERT and shelterin gene expression in young and older horses

**Fig. 2:**
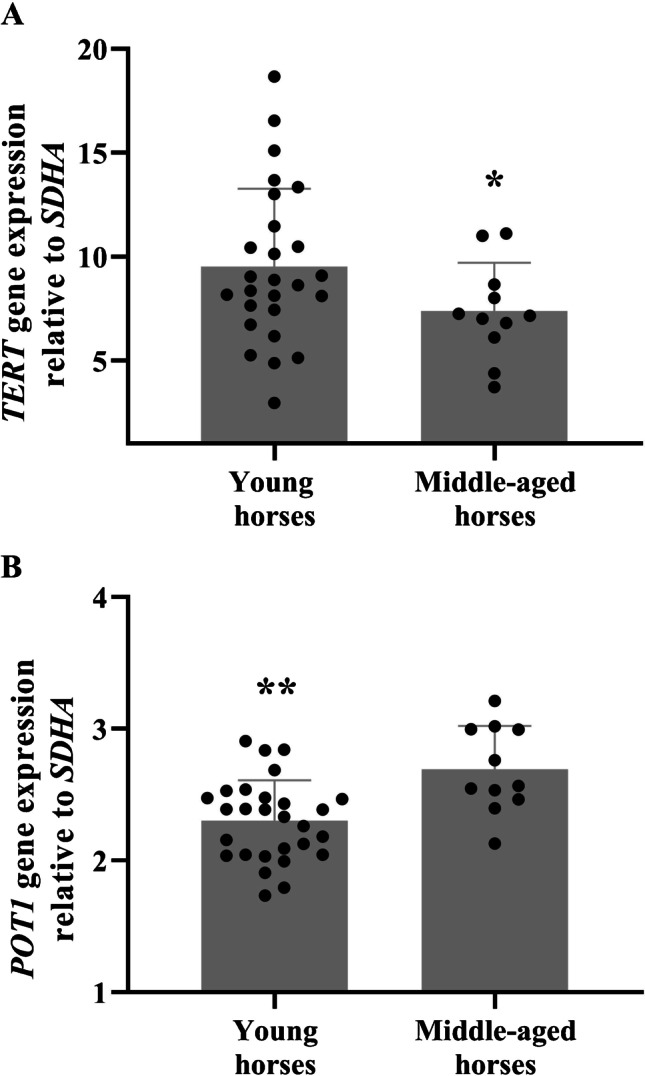
TERT and POT1 gene expression changes with ageing. Differentially expressed *TERT* (**A**) and *POT1* (**B**) gene expression differences in young versus middle-aged Thoroughbred racehorses. Data are mean ± SD relative gene expression. *p < 0.05, **p < 0.01

We then compared young (*n* = 29, 3.9 ± 0.28 years old) and middle-aged (*n* = 11, 14.8 ± 0.78 years old) horses’ *TERT* and shelterin gene expression at rest (before exercise) (Supplementary Table 6). The young horses were all actively training for racing, whilst the middle-aged horses were retired Thoroughbred racehorses. Compared to the young horses, middle-aged horses had reduced whole blood leukocyte *TERT* (*p* = 0.04) and elevated *POT1* (*p* = 0.002) gene expression (Fig. 2). No other shelterin genes were differentially expressed between young and middle-aged (retired) racehorses (all *p* > 0.05; Supplementary Table 6).

### Whole blood leukocyte microRNA expression after exercise training, and in young and older horses

**Fig. 3:**
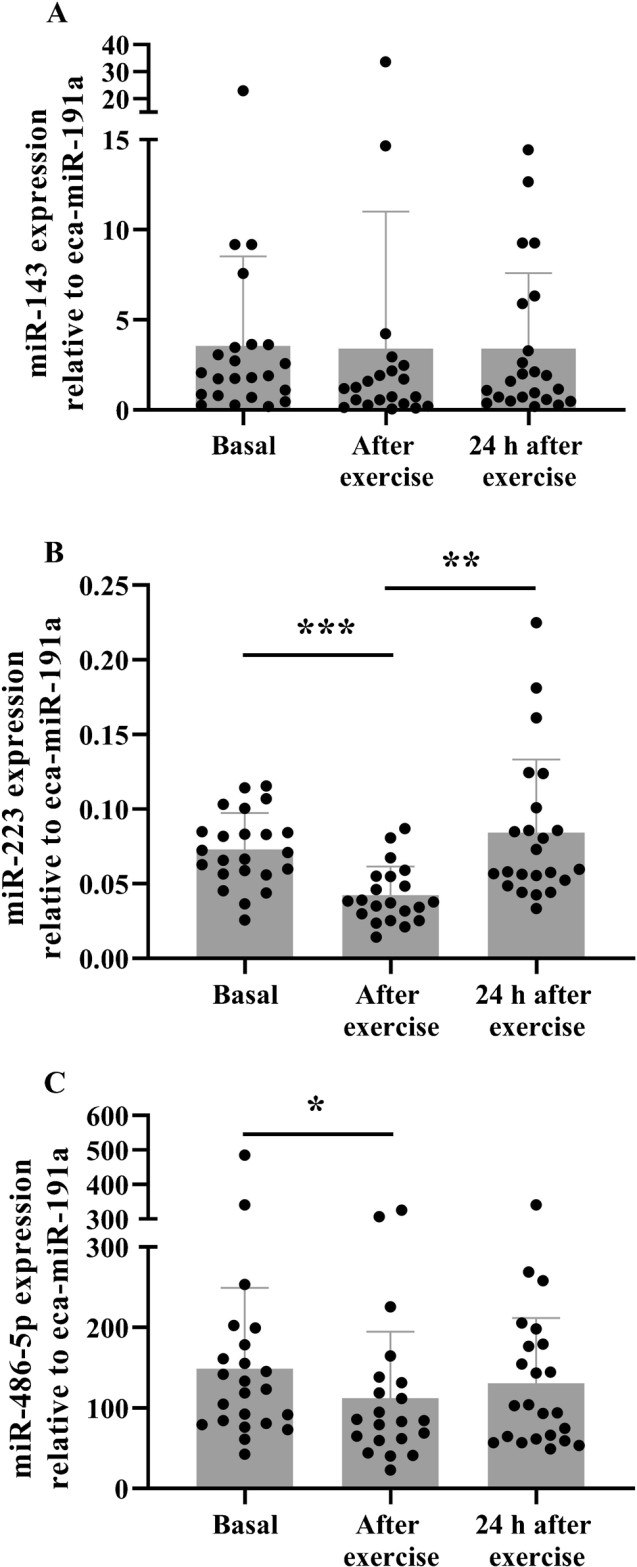
Leukocyte microRNA expression after exercise in Thoroughbred racehorses. Leukocyte microRNA expression changes caused by exercise training (**A–C**). Data are expressed as mean ± SD relative miRNA expression. *p < 0.05, **p < 0.01, ***p < 0.001

Whole blood leukocyte miR-223 and miR-486-5p expression were reduced immediately after exercise and returned to basal levels 24 h later (both *p* < 0.05; Fig. 3B  and C and Supplementary Table 5). Conversely, exercise did not alter whole blood leukocyte miR-143 expression (*p* > 0.05; Fig. 3A).

**Fig. 4:**
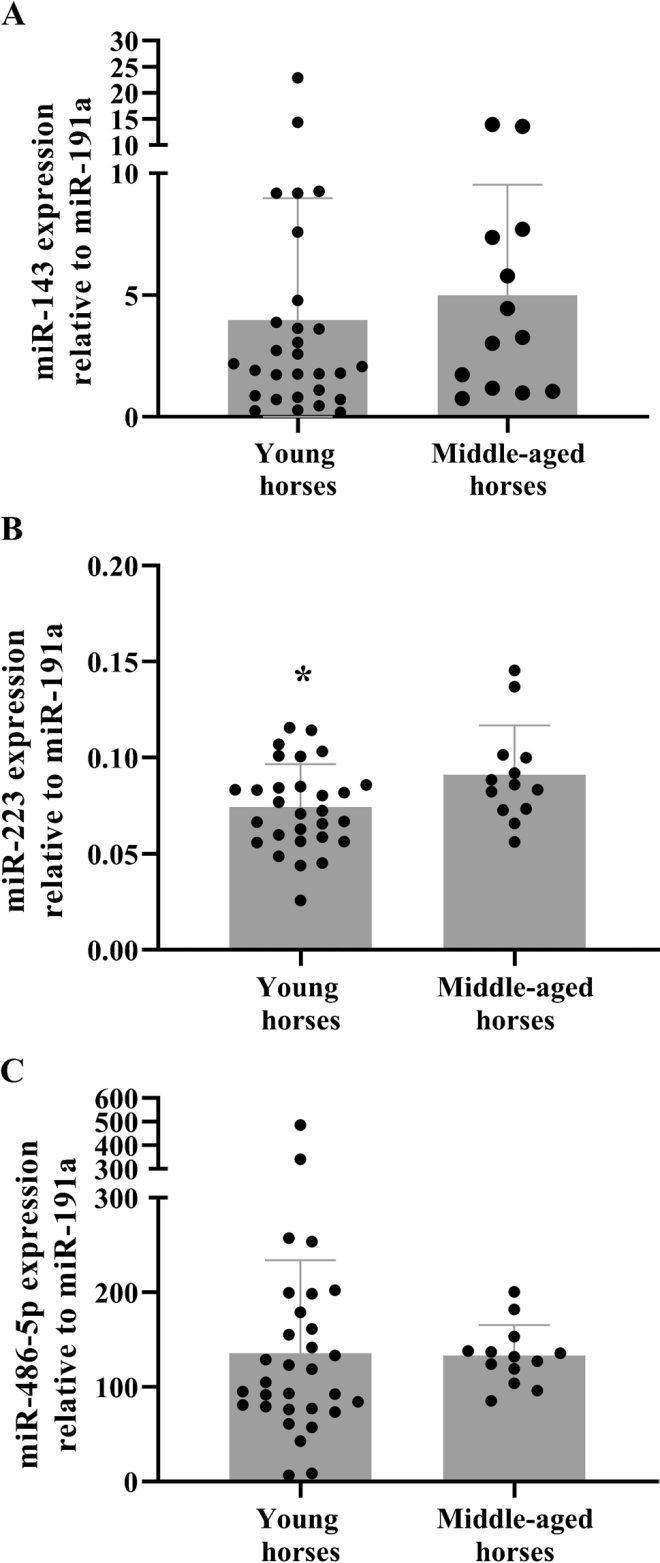
Leukocyte miRNAs and ageing. Leukocyte miR-143, miR-233 and miR-486-5p miRNAs in young and middle-aged Thoroughbred horses (**A**, **B** and **C**, respectively). Data are expressed as mean ± SD relative miRNA expression. *p < 0.05

Out of the three miRNAs analysed, only miR-223 was differentially expressed between young and older horses (Supplementary Table 6 and Fig. 4); it was significantly increased in older horses relative to their younger counterparts (*p* = 0.04).

## Discussion

Regular exercise training improves health span and may attenuate biological ageing through the preservation of telomeres. Current evidence suggests that exercise modulates *TERT* and telomerase activity, yet whether other molecules, such as miRNAs, have roles in telomere regulation is unclear. Here, we observed dynamic up-regulation of whole blood leukocyte *TRF1*, *TRF2* and *POT1* gene expression and concomitant decreases in miR-223 and miR-486-5p, immediately after a single bout of vigorous exercise, which returned to baseline levels 24 h after the training session in Thoroughbred racehorses. Furthermore, relative to the younger horses, retired — middle-aged — horses, not currently in training, exhibited significantly lower whole blood leukocyte *TERT* mRNA and elevated *POT1* mRNA and miR-223 expression. Therefore, a vigorous bout of exercise led to transient increases in key shelterin components and miRNAs implicated in biological ageing in a large, athletic mammal, the Thoroughbred racehorse.

Shelterin proteins control telomere length through interactions with DNA damage pathways, telomerase recruitment and regulating its activity. Whilst studies have indicated that a single bout of exercise, long-term training and athletic status are associated with increased *TERT* and telomerase activity in a variety of somatic cells, less is known on how exercise controls telomere-regulating genes (i.e. shelterin). It was previously demonstrated that *TERT* expression is increased 60 min after the cessation of intense aerobic exercise (30 min at 80% of peak oxygen uptake) in whole blood and sorted leukocytes (CD4^+^CD45RA^+^ and CD4^+^CD45RO^+^ T cells), and that this occurred with concomitant modulation of numerous telomere-regulating genes, including increased *TRF2IP* in humans [6]. Although whole blood leukocyte *TRF2IP* was not modulated after exercise here, leukocyte *TERT* expression was unchanged immediately after exercise, consistent with previous work [6]. However, we found that leukocyte *TRF1*, *TRF2* and *POT1* gene expression increased immediately after exercise and returned to baseline levels 24 h after, suggestive of an acute effect of exercise on shelterin that is likely to be telomere-protective.

TRF1, TRF2 and POT1 bind directly to the telomeres and control their length and integrity. TRF1 and TRF2 homodimerise and form protein duplexes to inhibit DNA damage/repair pathways and end-to-end fusions by binding exclusively to double-stranded telomeric DNA [39]. POT1 elongates telomeres via a telomerase-dependent mechanism and increases its processivity by interacting with ACD [7, 48]. POT1 also negatively regulates telomere length through inhibiting telomerase activity by binding to single-stranded telomeric repeats and via a TRF1-controlled mechanism [24]. The transient increase in leukocyte *TRF1*, *TRF2* and *POT1* gene expression after exercise may be a critical temporary response required to safeguard telomeres after physiological stress. Since long-term exercise training is associated with telomere maintenance in numerous rodent tissues [27, 49, 50] and long leukocyte telomeres in humans [31, 47], the shelterin responses identified after a single bout of exercise in the present study may play an important role in this process. This is, of course, speculative and will require further studies to experimentally verify.

The exercise training session in our study was a typical high-intensity workday. According to the GPS and heart rate data, racehorses were working at near-maximum intensity during the exercise session with average peak speeds of ~ 60 kph and heart rates of ~ 221 bpm, close to maximum heart rates reported elsewhere [4]. Our findings are in line with mRNA reports in rodent cardiac muscle after acute treadmill exercise (30-min bout at 70% of maximum running speed, at a 7° incline), with parallel increases in TRF1 and TRF2 protein content [28]. Long-term training studies in rodents suggest that TRF1 and TRF2 expression is increased in the heart and aorta in mice, and human endurance athletes have increased PBMC TRF1/TRF2 expression and telomerase activity [50]. Notably, PBMC *TRF1*, *TRF2* and *POT1* gene expression were increased 24 h after a 7-day ultra-marathon — sub-maximal but sustained, endurance exercise — in middle-aged human runners and this effect was not due to changes in the proportion of CD4^+^ and CD8^+^ cells [23]. A novel finding of our study was that whole blood leukocyte *TRF1*, *TRF2* and *POT1* gene expression was increased after exercise, but returned to baseline levels 24 h after training. Our data imply that any potential telomere protecting mechanisms are only transiently induced and regular training may be required for telomere maintenance in the long term.

Another novel observation of the present study was the age-related decrease and increase in whole blood leukocyte *TERT* and *POT1* gene expression, respectively. POT1 lengthens telomeres through a telomerase-dependent mechanisms in human cells [7] and enhances telomerase processivity by heterodimerising with ACD [48]. Thus, the increased *POT1* mRNA in the middle-aged horses may be an ineffective attempt to compensate for age-related telomere shortening, as they also had reduced *TERT* expression. TERT also exerts extra-telomeric effects, such that it controls exercise-induced changes to survival proteins regulating senescence in cardiovascular tissues [49, 50], and functions as a chromatin remodeller that influences transcriptional regulation [45]. Outside the nucleus, TERT improves glucose uptake in skeletal muscle via an insulin-insensitive pathway [40]. We could not elucidate whether the changes were exclusively age-related or potentially due to changes in exercise habits, since the middle-aged horses were retired and not currently in training for racing. Sedentary ageing controls are not available in this animal model, as Thoroughbred horses are bred to race. Aerobic exercise training is associated with increased *TERT* and telomerase activity in human PBMCs and rodent cardiovascular tissue [11]. A human study with young (~ 22 years old) and older adult (~ 71 years old) soccer players and sedentary age-matched controls did not report any statistically significant age-related differences in PBMC *POT1* mRNA, yet they observed increased telomerase activity in older adult controls relative to their younger counterparts [19]. Considering the relationship between *TERT* mRNA expression and telomerase activity, future work will be required to confirm our findings and establish a genuine age-related change or a decrease due to a lack of exercise training. Regardless of the underlying cause, therapeutic strategies such as *TERT* gene therapies may improve vitality and lifespan in horses, and, potentially, humans.

The second objective of the present study was to examine exercise-induced and ageing-associated changes in three miRNAs that are predicted to target *TERT* and shelterin mRNAs. To our knowledge, only one investigation has reported on the exercise-induced regulation of shelterin genes analysed in context with miRNAs [6]. They found that whole blood leukocyte miR-186 and miR-96, both predicted targets of *TRF2IP*, were significantly increased, whilst *TRF2IP* decreased 60 min after a 30-min bout of treadmill running at 80% of peak oxygen uptake in young men [6]. Here, we found that whole blood leukocyte miR-223 and miR-486-5p were both reduced immediately after vigorous exercise and returned to baseline levels 24 h after training, similar to the *TRF1*, *TRF2* and *POT1* mRNA responses. MiR-143 tended to be elevated immediately after exercise but did not reach statistical significance, possibly due to the marked inter-animal variation in gene expression after training. The acute exercise-induced decrease in whole blood leukocyte miR-486-5p in our study is congruent with previous findings in human whole blood [10], serum [2] and plasma [16]. Circulating miR-486-5p is also decreased by short-term aerobic and sprint interval training [2]. MiR-223 is increased in human plasma 1 h after an acute exercise [33] and is implicated in critical signalling pathways responsible for exercise-induced physiological adaptations [17]. Moreover, miR-223 is involved in exercise-induced physiological hypertrophy, as it was significantly increased in treadmill-trained mice after 4 weeks of training [52]. *TRF1* and *TRF2* are predicted targets of miR-486-5p, and both miR-486-5p and miR-223 target the 3′UTR of *POT1* according to the miRWalk database. Notably, these miRNAs were reduced immediately after exercise in conjunction with increases in their mRNA targets (*TRF1*, *TRF2* and *POT1*), suggesting that key shelterin genes are post-transcriptionally regulated by a single bout of vigorous training.

A significant increase in whole blood leukocyte miR-223 was also observed in middle-aged horses, compared to their younger counterparts, suggesting a role for this miRNA in ageing. That miR-233 is influenced by ageing is not a novel finding but is interesting considering the age-associated modulation of its target mRNA (*POT1*). For instance, plasma miR-223 is much higher in older adults (~ 75 years old), both healthy and physically active, as well as fragile older adults compared to young controls (~ 20 years old) [36]. However, the effects of ageing on miR-223 may be tissue-specific as it improves glucose control in the heart via Glut4 regulation [26]. Thus, future work should attempt to verify miR-223 targets *POT1* and its regulation in context with ageing across somatic cells.

It is important to note that the shelterin genes and miRNAs regulated by a single bout of vigorous exercise and differentially expressed in young and middle-aged animals in the present study were in Thoroughbred horses, who have been selectively bred for performance for centuries. Whilst it is tempting to speculate on the impacts selective breeding may have on exercise-induced responses in shelterin genes and miRNAs, it is reasonable to suggest that our findings must be interpreted in context with the genetic background. However, most of the literature deriving from rodent studies carries a similar concern. The molecular changes after exercise observed in the present study occurred in Thoroughbreds that were not direct relatives, yet 15 of the 29 horses shared a common great-grand-sire (*n* = 5) (i.e. two generations earlier). Five of the 11 middle-aged horses also had at least one common ancestor one or two generations prior. Considering that horses possess similar telomere lengths to that of humans [15], the lack of inbreeding in the horses used in the present study and the specific value of the animals as an athlete model, these findings may be more generalisable to humans than rodents.

There are some limitations associated with our work. Although we focused on the transcriptional regulation of shelterin genes via miRNAs, protein content was not analysed. Indeed, an increase in gene expression does not guarantee an increase in protein abundance. However, exercise-induced increases in shelterin mRNA and corresponding protein changes have been observed previously in human PBMCs [23]. Our analysis was restricted to the three time points and we may have missed other potential changes in *TERT*, shelterin genes and miRNAs at other periods. For example, whole blood leukocyte *TERT* is typically increased 1 h after acute exercise, rather than immediately after [6]. We did not control for diet and blood samples were collected from non-fasted animals. Thus, we cannot exclude dietary effects influencing the molecular findings in the present study. We could not differentiate between miRNAs that were bound to their mRNA targets and total mature miRNA levels, limiting our ability to suggest whether changes in miRNA levels were due to altered expression or due to their impacts on mRNAs. Nonetheless, we observed dynamic changes in key shelterin genes that return to baseline levels 24 h after training.

In summary, using a large, athletic animal model with telomere lengths much closer to those observed in humans, our findings suggest that key shelterin genes (*TRF1*, *TRF2* and *POT1*) and miR-223 and miR-486-5p are dynamically regulated by a single bout of vigorous exercise, yet they returned to baseline 24 h later. We also demonstrated age-related changes in key shelterin genes (*TERT* and *POT1*) and miR-223, which are all linked to biological ageing. Collectively, our data implicates miRNAs in the exercise- and ageing-induced modulation of shelterin, which may ultimately contribute to the molecular mechanisms responsible for the telomere maintenance associated with long-term training. Further work will be necessary to determine if long-term exercise training attenuates telomere attrition through regulation of shelterin/miRNAs and if *TERT*, shelterin or miRNA-based treatments can improve biological ageing in large mammals, such as horses and humans.

## Materials and methods

### Animals and sample processing

Horses from stables around Geelong and Melbourne (Victoria, Australia) were enrolled after written informed consent was obtained from owners or trainers. All animals were healthy male and female Thoroughbred racehorses either currently in training (young horses: *n* = 29 [15 females], 3.9 ± 1.5 years) or retired (older horses: *n* = 11 [5 females], 14.8 ± 2.6 years). All horses had raced multiple times. Based on survey data, the maximum reported lifespan of mixed breeds of horses provided with adequate care is 44 years, although few live past 30 [30]. Reports from Australia indicated that the median age of 974 horses aged 15 years or greater was 20.7 years (interquartile range: 17–23), of which 34% were Thoroughbreds [30]; specific longevity data from Thoroughbreds is not known given the costs associated with keeping them through their natural lifespan. Considering that the average life expectancy of humans from developed nations is approximately 80 years and some supercentenarians live over 110 years of age [1, 38], horses seem to age ~ 2.6 times quicker than humans. Thus, the young horses were broadly equivalent to human ten-year-olds, whilst the older horses were considered middle-aged. This study was approved by the University of New England’s Animal Ethics Committee and this research adhered to the Australian code for the care and use of animals for scientific purposes (NHMRC, Australia). Whole blood samples were collected from the jugular vein into PAXgene Blood RNA Tubes (BD Biosciences) and temporarily (~ 1–2 h) stored at room temperature before long-term storage at − 20 °C. Whole blood was collected at baseline, with horses in a rested condition, immediately after the exercise trial and 24 h after the exercise session.

### Exercise trial

Exercise trials were conducted in the morning (4–8 am) at stables located at Geelong and Caulfield racecourses, Victoria, Australia. Racehorse trainers were asked to instruct the jockeys riding the racehorses to perform a typical workday, which comprised a short warm (walking to trotting) before increasing the intensity to a canter, then a high-intensity bout of galloping. To determine the intensity of the session, a subset of the young horses (*n* = 13) were fitted with a wearable equine Global Positioning System (GPS) and heart rate monitor (E-Trakka System), which was used to monitor heart rate and speed during the exercise trial. The racehorses’ heart rates were recorded during exercise to illustrate the training intensities.

### TERT and shelterin gene expression

Gene expression was quantified using SYBR-based qPCR. Whole blood samples that were frozen at − 20 °C were thawed when preparing for RNA extraction. Using PAXgene Blood RNA Kit (PreAnalytiX), RNA was extracted from the whole blood samples. RNA was reverse transcribed using the High-Capacity cDNA Reverse Transcription Kit (ThermoFisher Scientific) and run on the Veriti 96-Well Thermal Cycler (Applied Biosystems). Ten-microliter reactions that comprised 2 × SensiFAST SYBR No-ROX (Bioline), 300 nM of forward and 300 nM reverse primers and ~ 10 ng of cDNA was run in triplicate with no template controls on the QuantStudio 7 Flex Real-Time PCR System (Applied Biosystems). The thermocycling conditions were the following: 95 °C for 2 min, followed by 40 cycles of 95 °C for 5 s and 58 to 60 °C for 20 s, followed by a melt curve (Supplementary Table 1). Only triplicates within 1 Ct were used in the analysis or the average of the duplicates was utilised. Gene expression was compared to the endogenous control, succinate dehydrogenase complex subunit A (*SDHA*), as it is an appropriate — stable — reference gene for exercise studies in horses [5]. Relative gene expression was calculated using the 2^−delta−delta^ Ct method.

### MicroRNA expression

RNA was extracted from PAXgene Blood RNA Tubes, checked by spectrometry and processed as outlined in the gene expression assays. Approximately 150 ng of RNA was reverse transcribed using the TaqMan MicroRNA Reverse Transcription Kit (ThermoFisher Scientific) as per the manufacturer’s guidelines. Five-microliter reaction that comprised 2 × SensiFAST Probe No-ROX (Bioline), 20 × TaqMan Assay (ThermoFisher Scientific), H_2_O and 5 ng of cDNA was run in triplicate on 384-well plates in the QuantStudio 7 Flex Real-Time PCR System (Applied Biosystems). The miRNA assay numbers are outlined in Supplementary Table 2. The cycling conditions were a hold at 50° for 2 min, a hold at 95° for 20 s, followed by 40 cycles of 95° for 1 s and then 60° for 20 s. The miRNA expression was compared to the endogenous control, miR-191a, as per a previous investigation [29]. miRNA expression was calculated using the 2^−delta−delta^ Ct method. Gene and miRNA data are expressed as relative expression.

### Statistical analyses

All statistical analyses and graphical representations were performed using GraphPad Prism (Version 9.0.2). Descriptive statistics were performed on the horse characteristics (age, sex and colour) and the details about their exercise session. A mixed-effect analysis was applied to the heart rate data. Mixed effect models were used to determine any changes in gene and miRNA expression observed after exercise training. Independent sample *t*-tests or Welch’s *t*-tests were used to examine differences in gene and miRNA expression between young and middle-aged horses. Data are expressed as mean ± SD and statistical significance was set at *p* < 0.05.

## Supplementary Information

Below is the link to the electronic supplementary material.

Supplementary file1 (DOCX 22 KB)
