# Supplementary material for: Exercise regulates shelterin genes and microRNAs implicated in ageing in Thoroughbred horses
Source: Pflugers Arch. 2022 Sep 9;474(11):1159–69. doi: 10.1007/s00424-022-02745-0 (PMC9560944; doi:10.1007/s00424-022-02745-0)
Supplement: Supplementary file 1 — Supplementary file1 (DOCX 22 KB) [file 424_2022_2745_MOESM1_ESM.docx]

**Supplemental Material**

Supplementary Table 1. Equine gene primer-sets.

| Gene symbol | Primer-sets | Amplicon (nt) | Ta (ºC) | Intra-assay CV (%) |
| --- | --- | --- | --- | --- |
| *TRF1* | F: GAC AAA CAG TCT GCG GTA ACT  R: AAT CTT GCT GTC GGC TTC C | 109 | 58 | 0.48±0.32 |
| *TRF2* | F: GTT CCT TTG ATA TGG AGG CTG A  R: CAC TGC CTC TGT CAG TGT AAA | 95 | 58 | 0.32±0.22 |
| *TRF2IP* | F: CCA CAG AAT AAG AGA ACT CCA GAC  R: ACT GCT TCT TCA TTC TCT TGG AT | 77 | 58 | 0.35±0.23 |
| *TINF2* | F: CAA AGC AGG ATC TGA GGA AGA T  R: TCG AAG CCA AAT CCA CAG G | 93 | 58 | 0.34±0.20 |
| *ACD* | F: CCT CGG ACT GGG AGG ATA A  R: GTC CAC CTG AAG GTA GAA CTC | 131 | 60 | 0.39±0.41 |
| *POT1* | F: TCA GAG GGT ATC TGT CCA TCA G  R: GTA GCC GAC AGT TGT TGA CAT | 107 | 58 | 0.26±0.15 |
| *TERT* | F: CAC GCT GCT CAC TCT ACC  R: GTG GTG CAG AGG TCA TAG AG | 82 | 60 | 0.42±0.31 |
| *SDHA* (control) | F: GTC CTG CAA CCT GG AGA TAA A  R: CGC AGT TCC GAC GTT CTT ATA C | 117 | 58 | 0.34±0.23 |

Legend: nt, nucleotides; Ta, annealing temperature; CV, coefficient of variation (intra-plate – triplicates); *TRF1*, telomeric repeat binding factor 1; *TRF2*, telomeric repeat binding factor 2; *TRF2IP*, *TRF2* interacting protein; *TIN2*, *TRF1* interacting nuclear factor 2; ACD, ACD shelterin complex subunit and telomerase recruitment; *POT1*, protection of telomeres 1; *SDHA*, succinate dehydrogenase complex flavoprotein subunit A.

Supplementary Table 2. MicroRNA experiments.

| MicroRNA | Assay number | Intra-assay CV (%) | mRNA target/location |
| --- | --- | --- | --- |
| hsa-miR-486-5p | 001278 | 0.85±0.55 | *POT1* (CDS and 3’UTR)  *TRF1* (3’UTR)  *TRF2* (CDS)  *TINF2* (3’UTR)  *TERT* (CDS)  *ACD* (CDS)  *TERF2IP* (CDS) |
| hsa-miR-223 | 002295 | 0.46±0.25 | *POT1* (3’UTR)  *TINF2* (CDS) |
| hsa-miR-143 | 002249 | 1.04±0.47 | *POT1* (CDS and 3’UTR)  *TINF2* (CDS and 3’UTR) |
| hsa-miR-191 (control) | 002299 | 0.68±0.42 |  |

TaqMan MicroRNA Assays from ThermoFisher Scientific. MiRNA-mRNA are from the miRWalk database. The intra-assay CV from miRNA experiments on triplicate samples are displayed as percentages.

Legend: CV, coefficient of variation; CDS, coding sequence; UTR, untranslated region; 3’, three prime; 5’, five prime.

Supplementary Table 3. Horse characteristics in the exercise trial

| **Variable (n = 23)** | **Mean ± SE** |
| --- | --- |
| Age (y) | 3.83 ± 0.325 |
| Sex (M/F/C/G/S) | 7/5/5/4/2 |
| Colour (Ba/C/Br/Bl) | 15/5/2/1 |

M, mare; F, filly; C, colt; G, gelding; S, stallion; Ba, Bay; C, chestnut; Br, brown; Bl, black.

Supplementary Table 4. Racehorse exercise session.

| **Variable (n = 13)** | **Mean ± SE** |
| --- | --- |
| Exercise duration (s) | 616 ± 40.5 |
| Peak speed (kph) | 60.3 ± 1.1 |
| Stride length at 50 kph (m) | 6.27 ± 0.05 |
| Peak heart rate (bpm) | 221 ± 2.64 |

s, seconds; m, metres; bpm, beats per minute.

Supplementary Table 5. Relative gene and miRNA expression after exercise.

| **Gene/microRNA** | **Basal**  **(n, mean ± SEM)** | **Immediately**  **after exercise**  **(n, mean ± SEM)** | **24 hour**  **after exercise**  **(n, mean ± SEM)** | **Main effect**  **(*p*-value)** |
| --- | --- | --- | --- | --- |
| *TERT* | 21, 9.33 ± .75 | 20, 7.99 ± .58 | 22, 9.185 ± 1.02 | .52 |
| *TRF1* | 22, 2.41 ± .09 | 20, 2.82 ± .08 | 22, 2.71 ± .125 | .01 |
| *TRF2* | 22, 8.73 ± 0.29 | 20, 9.72 ± 0.45 | 22, 9.20 ± .30 | .04 |
| *TINF2* | 22, 2.49 ± .14 | 20, 2.60 ± .25 | 22, 2.43 ± .17 | .71 |
| *ACD* | 18, 2.59 ± .18 | 19, 2.69 ± .26 | 20, 2.39 ± .14 | .43 |
| *POT1* | 22, 2.325 ± .07 | 20, 2.56 ± .09 | 22, 2.34 ± .09 | .02 |
| *TRF2IP* | 22, 2.475 ± .08 | 20, 2.635 ± .08 | 22, 2.54 ± .09 | .10 |
| miR-143 | 23, 3.56 ± 1.035 | 21, 3.41 ± 1.66 | 23, 3.41 ± 0.87 | .70 |
| miR-223 | 23, 0.07 ± 0.005 | 21, 0.04 ± 0.004 | 23, 0.08 ± 0.01 | .0007 |
| miR-486 | 23, 149.0 ± 20.92 | 21, 112.1 ± 18.0 | 23, 130.8 ± 16.89 | .009 |

Supplementary Table 6. Relative gene and miRNA expression in young and retired, middle-aged horses.

| **Gene/microRNA** | **Young**  **horses**  **(n, mean ± SEM)** | **Middle-aged**  **horses**  **(n, mean ± SEM)** | ***p*-value** |
| --- | --- | --- | --- |
| *TERT* | 26, 9.51 ± .74 | 11, 7.38 ± .70 | .04 |
| *TRF1* | 29, 2.37 ± .12 | 11, 2.32 ± .10 | .76 |
| *TRF2* | 28, 8.63 ± .25 | 11, 9.21 ± .26 | .11 |
| *TINF2* | 29, 2.56 ± .16 | 11, 2.62 ± .18 | .86 |
| *ACD* | 23, 2.65 ± .15 | 9, 2.75 ± .22 | .695 |
| *POT1* | 29, 2.22 ± .10 | 11, 2.69 ± .10 | .002 |
| *TRF2IP* | 28, 2.50 ± .09 | 11, 2.39 ± .05 | .27 |
| miR-143 | 29, 3.98 ± 0.93 | 13, 4.98 ± 1.26 | .52 |
| miR-223 | 29, 0.07 ± 0.004 | 13, 0.09 ± 0.007 | 0.04 |
| miR-486 | 31, 135.6 ± 17.66 | 13, 133.4 ± 8.83 | .94 |
